# Accuracy of advanced versus strictly conventional 12-lead ECG for detection and screening of coronary artery disease, left ventricular hypertrophy and left ventricular systolic dysfunction

**DOI:** 10.1186/1471-2261-10-28

**Published:** 2010-06-16

**Authors:** Todd T Schlegel, Walter B Kulecz, Alan H Feiveson, E Carl Greco, Jude L DePalma, Vito Starc, Bojan Vrtovec, M Atiar Rahman, Michael W Bungo, Matthew J Hayat, Terry Bauch, Reynolds Delgado, Stafford G Warren, Tulio Núñez-Medina, Rubén Medina, Diego Jugo, Håkan Arheden, Olle Pahlm

**Affiliations:** 1 Human Adaptation and Countermeasures Division, NASA Johnson Space Center, Houston, TX, USA; 2 Department of Electrical Engineering, Arkansas Tech University, Russellville, AR, USA; 3 Department of Engineering, Colorado State University, Pueblo, CO, USA; 4 Institute of Physiology, School of Medicine, University of Ljubljana, Ljubljana, Slovenia; 5 Division of Cardiovascular Medicine, University of Texas Health Science Center, Houston, TX, USA; 6 Biostatistics, School of Nursing, Johns Hopkins University, Baltimore, MD, USA; 7 Division of Cardiology, University of Texas Health Science Center, San Antonio, TX, USA; 8 Heart and Lung Treatment and Transplant Center, Texas Heart Institute, Houston, TX, USA; 9 Cardiac Catheterization Laboratory, Thomas Memorial Hospital, Charleston, WV, USA; 10 Instituto de Investigaciones Cardiovasculares, Universidad de los Andes, Mérida, Venezuela; 11 Grupo de Ingenieria Biomedica, Universidad de Los Andes, Mérida, Venezuela; 12 Department of Clinical Physiology, Lund University Hospital, Lund, Sweden

## Abstract

**Background:**

Resting conventional 12-lead ECG has low sensitivity for detection of coronary artery disease (CAD) and left ventricular hypertrophy (LVH) and low positive predictive value (PPV) for prediction of left ventricular systolic dysfunction (LVSD). We hypothesized that a ~5-min resting 12-lead *advanced *ECG test ("A-ECG") that combined results from both the advanced and conventional ECG could more accurately screen for these conditions than strictly conventional ECG.

**Methods:**

Results from nearly every conventional and advanced resting ECG parameter known from the literature to have diagnostic or predictive value were first retrospectively evaluated in 418 healthy controls and 290 patients with imaging-proven CAD, LVH and/or LVSD. Each ECG parameter was examined for potential inclusion within multi-parameter A-ECG scores derived from multivariate regression models that were designed to optimally screen for disease in general or LVSD in particular. The performance of the best retrospectively-validated A-ECG scores was then compared against that of optimized pooled criteria from the strictly conventional ECG in a test set of 315 additional individuals.

**Results:**

Compared to optimized pooled criteria from the strictly conventional ECG, a 7-parameter A-ECG score validated in the training set increased the sensitivity of resting ECG for identifying disease in the test set from 78% (72-84%) to 92% (88-96%) (P < 0.0001) while also increasing specificity from 85% (77-91%) to 94% (88-98%) (P < 0.05). In diseased patients, another 5-parameter A-ECG score increased the PPV of ECG for LVSD from 53% (41-65%) to 92% (78-98%) (P < 0.0001) without compromising related negative predictive value.

**Conclusion:**

Resting 12-lead A-ECG scoring is more accurate than strictly conventional ECG in screening for CAD, LVH and LVSD.

## Background

Although conventional resting electrocardiography (ECG) has an important role in managing acute coronary syndromes and suggestive but non-diagnostic acute chest pain, it has well-recognized limitations in the detection of heart disease[1]. For both isolated and pooled ECG abnormalities, the sensitivity of conventional resting ECG as a predictor for coronary artery disease (CAD) and left ventricular hypertrophy (LVH) has been too low for it to be practical as a screening tool[2,3]. Although conventional resting ECG, when normal, has excellent negative predictive value (NPV) for left ventricular systolic dysfunction (LVSD), the simultaneously poor positive predictive value (PPV) of abnormal ECG findings also limits conventional ECG's utility in heart failure screening[4,5]. Thus, any improvements to the resting ECG that might notably increase its sensitivity for identifying CAD and LVH (without compromising related specificity) and/or its PPV in screening for LVSD (without compromising related NPV) would be clinically relevant.

Over the past two decades, several advanced techniques implemented within software have improved the diagnostic and/or predictive value of resting ECG. These techniques have included derived "3-dimensional" (spatial/spatiotemporal) ECG;[6-9] high-frequency (HF) QRS ECG;[10] detailed studies of waveform complexity by singular value decomposition (SVD); [8,11-13] and beat-to-beat QT variability (QTV) [14-17] and R-wave to R-wave variability (RRV)[18,19]. A theoretical advantage of computerized ECG systems is that they allow for multiple conventional and advanced ECG techniques to be performed in software during a single digital recording. Related results can then be integrated automatically by using statistical pattern recognition techniques [20] to maximize diagnostic or predictive accuracy. In practice, these procedures can also be performed rapidly and relatively inexpensively.

The hypotheses of the present study were that a ~5-min resting 12-lead advanced ECG test ("A-ECG"), defined as the multivariate logistical integration of key results from both the conventional and advanced ECG, could detect common cardiac conditions such as CAD and concentric LVH with greater sensitivity and accuracy than optimized pooled criteria from the strictly conventional ECG and also predict LVSD with greater PPV and accuracy.

## Methods

### Participants

Data from all individuals who volunteered for resting ~5-min high-fidelity ECG studies from 2001 through mid-2007 (training set) or thereafter (test set) were considered for inclusion. These included data from: *1*) Cardiac clinic patients who volunteered for individual studies at any of the following clinical sites: Texas Heart Institute (Houston, TX); the University of Texas Medical Branch (Galveston, TX); the University of Texas Health Sciences Center (San Antonio, TX); Brooke Army Medical Center (San Antonio, TX); St. Francis Hospital (Charleston, WV); the Universidad de los Andes (Mérida, Venezuela); and Lund University Hospital (Lund, Sweden); and *2*) Asymptomatic individuals who volunteered as "controls" at any of the following sites: Johnson Space Center (Houston, TX); the Universidad de los Andes and Lund University Hospital. For the test set, additional data from patients whose ~5-min ECGs had been collected at the Charleston Area Medical Center as part of earlier studies but that became available to us during 2007 (i.e., the STAFF III database)[7] were also utilized. All participants gave original informed consent, and the Institutional Review Boards of one or more of the institutions approved the studies.

### Inclusion criteria

For both the training and the test sets, to define our "Disease" groups, we included data only from those cardiac clinic patients whose disease (CAD, LVH and/or LVSD) was proven based on ECG-independent information derived from standard clinical imaging tests [16,21-23] performed within one month of ECG testing by investigators or other clinicians blinded to the automatically-produced A-ECG results. Disease was defined as the presence of at least one of the following: *1*) CAD, defined as a coronary angiogram showing at least one obstruction ≥50% in at least one major native coronary vessel or coronary graft, or, if for clinical reasons angiography was not performed, then one or more reversible perfusion defects on 99 m (Tc)-tetrofosmin single-photon emission computed tomography (SPECT); [16,21,23]*2*) LVH, defined as moderate or greater concentric hypertrophy or concentric remodeling according to the guidelines of the American Society of Echocardiography;[24] and/or *3*) LVSD of any etiology, defined as LVEF <50% by echocardiography, cardiac magnetic resonance imaging (CMR) or SPECT. Diseased individuals who met none of these three inclusion criteria but who had isolated right ventricular pathology, isolated LV diastolic dysfunction, isolated LV cavity enlargement or isolated fixed defect on SPECT were excluded from the study.

To derive correspondingly definitive "Healthy" groups for both the training and test sets, we included data only from low-risk asymptomatic controls who had no evidence of cardiovascular or other systemic disease based on a negative history and physical examination. Asymptomatic controls who were hypertensive (BP≥140/90), receiving treatment for hypertension, diabetic or active smokers were excluded. All cardiac clinic patients or asymptomatic individuals who had complete bundle branch block, sinus tachycardia, non-sinus rhythm, paced rhythm, pre-excitation, or an incomplete ECG recording were also excluded from both the training and test sets.

### Training set

Of the 952 individuals who were considered for the training set, 708 met the above inclusion criteria, including 290 for the Disease group training set and 418 for the Healthy group training set. Of the 290 patients constituting the Disease group training set, 188 had normal LV function (136 had CAD; 25 had LVH; and 27 had both CAD and LVH) and constituted a "Disease without LVSD" training subset, whereas another 102 had LVEF <50% (77 with ischemic cardiomyopathy; 25 with nonischemic dilated cardiomyopathy) and constituted a "Disease with LVSD" training subset. Of the 418 controls in the Healthy group training set, a majority also had their disease-free status further demonstrated through normal or unremarkable results on a conventional or SPECT exercise stress test, echocardiogram, and/or CMR test performed for research purposes within 2 years of their ~5-min ECG. These included 55 elite, endurance-trained normotensive Swedish athletes (38 males) who had had clinically unremarkable CMR results.

### Test set

Data for the test set were obtained from an additional 315 individuals, including from an additional 208 diseased patients and an additional 107 healthy controls. The 208 individuals in the Disease group test set consisted of 139 patients with CAD, 17 with concentric LVH, 11 with both CAD and LVH, and 41 with LVSD (27 with ischemic and 14 with nonischemic dilated cardiomyopathy). The Healthy group test set consisted of 107 consecutive individuals over age 35 (including 9 elite athletes) who met the Healthy group inclusion criteria, recruited after mid-2007 mainly at NASA's Human Test Subject Facility in Houston. Within the Disease Group test set, data for 97 of the 208 patients came from the pre-procedural portion of the STAFF III database[7]. Since all patients in the STAFF III database had catheterization-proven CAD but unreported LV function, their data, as well as data from another 26 diseased patients with unknown LV function were by necessity withheld from the LVSD-related sub-analyses in the test set.

### ECG data collection and analyses

At all sites, a high-fidelity (1000 samples/sec) computerized 12-lead ECG system (Siemens-Elema AB, Solna, Sweden or CardioSoft, Houston, TX) was used to acquire at least 256 waveforms acceptable for signal averaging and variability analyses.

### A. Conventional ECG parameters and criteria

Signals from the first 10 sec of the conventional ECG recording were analyzed automatically in software to quantify all major intervals, axes, and voltages as well as ST segment levels. Initial candidate criteria used for defining these strictly conventional 12-lead ECGs as "abnormal" were: *1*) LVH according to traditional Sokolow-Lyon voltage criteria (SV1 + RV5 or RV6 ≥3.5 mV) or to gender-specific Cornell voltage (RaVL + SV3 ≥2.8 mV in men or ≥2.0 mV in women) or Cornell product (244 mV*ms with a 0.8 mV adjustment for women) criteria;[25]*2*) old infarction according to Anderson et al's subset of Selvester's criteria;[26]*3*) resting ST depressions or T-wave abnormalities according to computerized Minnesota Codes 4.1 to 4.4 and 5.1 to 5.3; *4*) prolonged QTc (≥450 ms in men and ≥460 ms in women) or QRS (>110 ms) interval (individuals with complete bundle branch blocks being excluded from the study); or *5*) left axis deviation (≤-30°).

### B. Advanced ECG parameters obtained after signal averaging

Signal averaging was performed over the entire ~5-min (256-beat) recording using software developed by the authors[10,13] to generate results for parameters of: *1*) 12-lead HF QRS ECG;[10]*2*) derived 3-dimensional ECG, using the regression-related Frank-lead reconstruction technique of Kors et al[27] to generate several vectocardiographic parameters, including for example the spatial mean QRS-T angle,[6,8,28] the spatial maximums ("peaks") QRS-T angle[9] and the magnitude, [28] direction[28] and beat-to-beat variation[29] of the spatial ventricular gradient and its components; and *3*) QRS and T-waveform complexity via SVD, to derive for example the principal component analysis (PCA) ratio,[11,13,30] the relative residuum[12,13] and the dipolar and nondipolar voltage equivalents[8] of the QRS and T waveforms. The majority of these parameters and their related detailed methods have been described in other recent publications[10,13,31]. We also generated results for several other potentially promising parameters (see Additional file 1: Supplemental Table 1 for partial list), including, for example, for the spatial ventricular activation time [32] and the total integral of the Z-lead QRS complex above 5 Hz ("Z integral")[33].

### C. Advanced parameters derived from variability analyses

Several parameters of 256-beat RRV and QTV described in previous publications[17,31,34] were again evaluated via custom software programs[17]. These included the QT variability index (QTVI), but using the means and variances of the RR interval[15] rather than those of the heart rate[14] in the denominator of the QTVI equation, and the "unexplained" part of QTV[31,34].

### Statistical Analyses (including generation, validation and testing of A-ECG scores)

Using the training set, promising candidate subsets of ECG parameters for potential inclusion in primary ("Healthy versus Disease") and secondary ("Disease with versus without LVSD") A-ECG scores were first identified using a branch-and-bound feature selection procedure [20] implemented in SAS 9.1.3 (Cary, NC). To avoid the so-called "curse of dimensionality", the number of ECG parameters incorporable into any potential A-ECG score was limited to fewer than one-tenth of the minimum number of training samples available in a given group or subgroup[20]. Logistic regression was used to retrospectively estimate the probability of any subject in the training set being a member of the Disease group, and of any diseased subject in the training set being a member the "Disease with LVSD" subgroup, based strictly on his/her A-ECG-based independent variables and a cutoff predicted probability of >0.5. The best candidate subsets of parameters (A-ECG scores) were then further validated by bootstrap analysis[35] in which for each fixed score, the data were iteratively resampled 1000 times and the logistic regression coefficients for each parameter in the given score re-estimated. The bootstrap analyses, implemented in Stata 10.0 (College Station, TX), revealed not only the variability in the coefficients, but also those candidate A-ECG scores that should be discarded because of their doubtful utility for classifying later subjects in the test set, for example scores with coefficients that varied greatly or that did not have the expected sign over all 1000 bootstraps. Prior to subsequent evaluation in the test set, the bootstrap-validated A-ECG scores were further evaluated within the training set via a jackknife procedure[35] in which the score's sensitivity, specificity, accuracy or predictive values were assessed by using the data for all but one observation in the training set to classify the omitted observation, then repeating the process for each observation in turn. Comparisons of accuracies (sensitivities and specificities) and predictive values between strictly conventional and A-ECG classifiers were performed using Cochran's Q[36] and Wald tests, respectively, the latter employing the difference-based weighted least squares method[37]. For simple illustrative comparisons between groups, the Wilcoxon rank sum and receiver operating curve characteristic statistics were used.

## Results

Table 1 shows baseline characteristics of the Disease and Healthy groups and of the two Disease subgroups in the training set. Besides being free of hypertension and diabetes and having lower body mass index and medication use, the Healthy group training set also was younger than the Disease group training set. Because of the age disparity, two sets of primary A-ECG scores were constructed and validated in the training set for later evaluation in the test set: one wherein all healthy subjects were included and one wherein only those healthy subjects >40 years of age (mean 51 ± 8 years, N = 133, 63% men) were included. For the additional 315 individuals who comprised the test set, the distributions of hypertension, diabetes, body mass index, LVEF, and medication use were similar to those shown in Table 1. The mean ages in the test set were 59 ± 12, 59 ± 13, 56 ± 12 and 49 ± 11 years for the Disease group, the with-LVSD and without-LVSD Disease subgroups and the Healthy group respectively, men comprising 65%, 74%, 60% and 57% of those groups, respectively.

**Table 1:** Demographic Characteristics of the Training Set Disease Group, Disease Subgroups and Healthy Group

Parameter	Disease Group (N = 290)	Disease Subgroup A With LVSD (LVEF < 50%, N = 102)	Disease Subgroup B Without LVSD (LVEF≥50%, N = 188)	Healthy Group (N = 418)
Age [years]	59 ± 11	56 ± 13	60 ± 10	36 ± 12
Males	189 (65)	72 (71)	117 (62)	255 (61)
BMI [kg/m^2^]	29 ± 7	29 ± 7	29 ± 6	26 ± 4
LVEF (%)	48 ± 16	29 ± 10	59 ± 8	NA
Diabetes	111 (38)	32 (31)	79 (42)	0 (0)
Hypertension	116 (40)	30 (29)	86 (46)	0 (0)
Beta blockers	222 (77)	85 (83)	137 (73)	4 (1)
ACEIs or ARBs	127 (44)	42 (41)	85 (45)	0 (0)
Nitrates	104 (36)	30 (29)	74 (39)	0 (0)
Diuretics	130 (45)	79 (77)	51 (27)	0 (0)
Inotropes	50 (17)	44 (43)	6 (3)	0 (0)

Values are mean ± standard deviation for age, BMI (body mass index) and left ventricular ejection fraction (LVEF). All other values represent total number (percent) of affected individuals. NA, not applicable; ACEIs, angiotensin converting enzyme inhibitors; ARBs, angiotensin receptor blockers.

**Figure 1:**
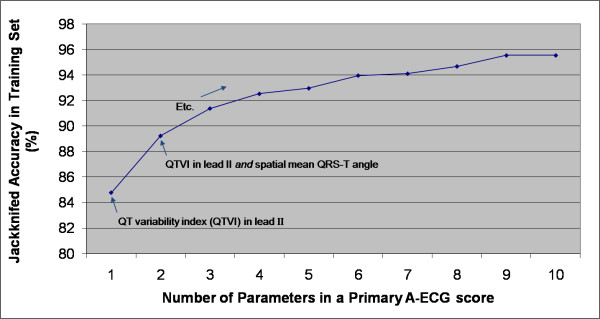
**Effect of number of parameters in a primary ("Healthy versus Disease") Advanced ECG (A-ECG) score on the score's jackknifed accuracy in the training set (N = 708)**.

**Figure 2:**
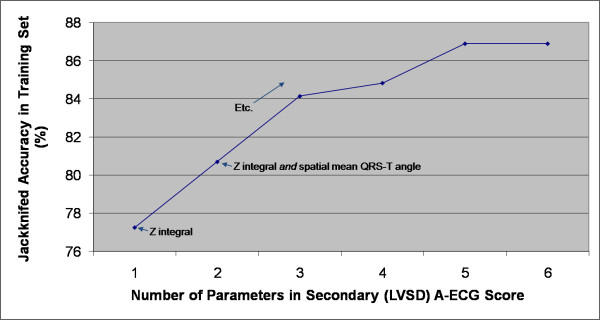
**Effect of the number of parameters in a secondary ("Disease with versus without left ventricular systolic dysfunction", LVSD) Advanced ECG (A-ECG) score on the score's jackknifed accuracy in the training set (N = 290)**.

Figures 1 and 2 show how the performance of an A-ECG score in the training set depended on the number of ECG parameters the score incorporated. For primary ("Healthy versus Disease") A-ECG scores (Figure 1, N = 708), only negligible further gains in cross-validated accuracy occurred with scores containing more than ~9 parameters. For secondary ("Disease with versus without LVSD") A-ECG scores (Figure 2, N = 290), this same cutoff occurred at only ~5 parameters. The first and second parameters incorporated into primary A-ECG scores by the automatic selection procedures were the QTVI in lead II and the spatial mean QRS-T angle, respectively (Figure 1). For secondary (LVSD) A-ECG scores, the first and second parameters incorporated by the same procedures were the Z integral and the spatial mean QRS-T angle, respectively (Figure 2).

Table 2 shows the performances *in the training set *of the pooled, strictly conventional ECG criteria, along with those of the most relevant single parameters and A-ECG scores. The candidate conventional ECG criteria outlined in the Methods section were retrospectively optimized when their Sokolow-Lyon subcriteria were dropped and replaced instead by subcriteria for left atrial abnormality (P-wave duration >120 ms or terminal negative component of a biphasic P-wave in lead V_1 _>4 ms*mV in area). Thus, only the resulting optimized set of conventional ECG criteria was carried forward for later use with the test set. Not unexpectedly, the retrospectively optimized A-ECG scores outperformed the retrospectively optimized pooled conventional ECG criteria in the training set. Of note, the optimal primary A-ECG scores made use of the entire ~5-min (so called "full-disclosure") 12-lead recording because they incorporated results from QTVI (Figure 1). Inasmuch as most 12-lead ECG machines do not yet have full-disclosure capabilities, Table 2 also shows the diagnostic performance in the training set of an optimized primary A-ECG score that was only allowed to incorporate results from parameters likely yielding reliable and reproducible results within strictly "snapshot" (10-sec) ECG recordings.

**Table 2:** Accuracies and Predictive Values of Pooled Conventional versus A-ECG Criteria *in the Training Set*

	Disease (N = 290)		Healthy (N = 418)					
	**TP**	**FN**	**TN**	**FP**	**Sensitivity(CLs)**	**Specificity(CLs)**	**Accuracy(CLs)**	

***Conventional ECG status***								
Abnormal (nominal pooled criteria)	221	69	352	66	76%(71-81%)	84%(80-88%)	81%(78-84%)	
Abnormal (optimized pooled criteria)	223	67	368	50	77%(72-82%)	88%(85-91%)	83%(81-86%)	
***Best individual parameter status***								
Abnormal QTVI in lead II (>-1.64 units)	242	48	359	59	83%(79-88%)	86%(82-89%)	85%(82-87%)	
***Best 1° A-ECG scores status***								
*"Full Disclosure" (5-min) A-ECG:*								
Abnormal 9-parameter score*	273	17	402	16	94%(91-97%)‡	96%(94-98%)‡	95%(94-97%)‡	
Abnormal 7-parameter score*	268	22	398	20	92%(89-95%)‡	95%(93-97%)‡	94%(92-95%)‡	
*"Snapshot" (10-sec) A-ECG:*								
Abnormal 7-parameter score	258	32	395	23	89%(85-92%)‡	94%(92-96%)‡	92%(90-94%)‡	
	**Disease** **+LVSD** (N = 102)		**Disease** **no LVSD** (N = 188)					
	**TP**	**FN**	**TN**	**FP**	**PPV (CLs)**	**NPV (CLs)**	**+LR**	**-LR**
*Conventional ECG status*								
Abnormal (nominal pooled criteria)	92	10	60	128	42%(35-49%)	86%(75-93%)	1.32	0.31
Abnormal (optimized pooled criteria)	94	8	59	129	42%(36-49%)	88%(78-95%)	1.34	0.25
***Best individual parameter status***								
Abnormal Z integral (>12.4 mV*ms)	77	25	153	35	69%(59-77%)†	86%(80-91%)	4.05	0.30
***Best 2° A-ECG scores status***								
Abnormal 5-parameter score	79	23	173	15	77%(68-85%)‡	92%(87-95%)	9.71	0.25

See text for definitions of nominal versus optimized pooled criteria for "abnormal" conventional ECGs. A-ECG, advanced ECG; LVSD, left ventricular systolic dysfunction; TP and FP, true and false positives; TN and FN, true and false negatives; CLs, exact 95% binomial confidence limits; PPV and NPV, positive and negative predictive values; +LR and -LR, positive and negative likelihood ratios; QTVI, QT interval variability index; Z integral, the total integral of the Z-lead QRS complex above 5 Hz.

*The 9-parameter 1° A-ECG score performed best in the training set whereas the 7-parameter 1° A-ECG score ultimately performed best in the test set (Table 3). All results shown for A-ECG scores are jackknifed.

†P < 0.05 and ‡P < 0.0001 versus the optimized pooled conventional ECG criteria.

Table 3 shows the performances *in the test set *of the optimized pooled conventional ECG criteria and of the most relevant single parameters and A-ECG scores generated in the training set. Although as expected most A-ECG scores tended to have slightly diminished performance in the test set compared to the training set (compare Table 3 to Table 2), several primary A-ECG scores generated from the training set still had accuracies of 90% or greater in the test set. For example, compared to the optimized pooled criteria from the strictly conventional ECG, the best 7-parameter primary full-disclosure A-ECG score generated in the training set increased the sensitivity of resting ECG for identifying Disease in the test set from 78% (72-84%) to 92% (88-96%) (P < 0.0001) while also increasing specificity from 85% (77-91%) to 94% (88-98%) (P < 0.05). Another 7-parameter A-ECG score that only incorporated parameters likely yielding reliable and reproducible results within "snapshot" ECG recordings was only slightly less accurate. In diseased patients, another 5-parameter *secondary *A-ECG score generated in the training set also increased the PPV of ECG for additionally predicting LVSD in the test set from 53% (41-65%) to 92% (78-98%) (P < 0.0001) without significantly compromising NPV. This secondary A-ECG score had corresponding positive and negative likelihood ratios for LVSD in the test set of 12.16 and 0.18, respectively, versus 1.23 and 0.21 for the optimized pooled conventional ECG criteria. The exact components and coefficients of those training set-generated primary and secondary A-ECG scores that performed best in the test set are shown in Additional file 2 (Supplemental Table 2).

**Table 3:** Accuracies and Predictive Values of Pooled Conventional versus A-ECG Criteria *in the Test Set*

	Disease (N = 208)		Healthy (N = 107)					
	TP	FN	TN	FP	Sensitivity(CLs)	Specificity(CLs)	Accuracy(CLs)	
*Conventional ECG status*								
Abnormal (optimized pooled criteria)	163	45	91	16	78%(72-84%)	85%(77-91%)	81%(76-85%)	
Best individual parameter status*								
Abnormal QTVI in lead II (>-1.64 units)	161	47	87	20	77%(71-83%)	81%(73-88%)	79%(74-83%)	
Best 1° A-ECG scores status* "Full Disclosure" (5-min) A-ECG:								
Abnormal 9-parameter score	185	23	98	9	89%(84-93%)‡	92%(85-96%)	90%(86-93%)†	
Abnormal 7-parameter score	194	16	101	6	92%(88-96%)‡	94%(88-98%)†	93%(90-96%)†	
*"Snapshot" (10-sec) A-ECG:*								
Abnormal 7-parameter score	192	16	92	15	92%(88-96%)‡	86%(78-92%)	90%(86-93%)†	
	**Disease** **+LVSD** (N = 41)		**Disease** **no LVSD** (N = 44)					
	**TP**	**FN**	**TN**	**FP**	**PPV (CLs)**	**NPV (CLs)**	**+LR**	**-LR**
*Conventional ECG status*								
Abnormal (optimized pooled criteria)	39	2	10	34	53%(41-65%)	83%(51-98%)	1.23	0.21
Best individual parameter status*								
Abnormal Z integral (>12.4 mV*ms)	28	13	31	13	68%(52-82%)	70%(55-83%)	2.31	0.45
Best 2° A-ECG scores status*								
Abnormal 5-parameter score	34	7	41	3	92%(78-98%)‡	85%(72-94%)	12.16	0.18

A-ECG, advanced ECG; LVSD, left ventricular systolic dysfunction; TP and FP, true and false positives; TN and FN, true and false negatives; CLs, exact 95% binomial confidence limits; PPV and NPV, positive and negative predictive values; +LR and -LR, positive and negative likelihood ratios; QTVI, the QT interval variability index; Z integral, the total integral of the Z-lead QRS complex above 5 Hz.

*The best individual parameters and their cut-off values and the best A-ECG scores are all carried over from the training set and are not necessarily fully optimized with respect to the test set.

†P < 0.05 and ‡P < 0.0001 versus the optimized pooled conventional ECG criteria.

## Discussion

The results of this study suggest that resting 12-lead A-ECG tests can detect the presence of catheterization-proven or other imaging-proven CAD and LVH with higher sensitivity and specificity than optimized pooled criteria from the strictly conventional ECG. This improved detection is accomplishable via the use of optimal combinations of 7 or fewer advanced and conventional ECG parameters within computerized multivariate A-ECG scores. Similar A-ECG scores can also increase the PPV of resting ECG for predicting LVSD without compromising related NPV.

Beginning in the 1960s, Pipberger et al applied a multivariate approach to the conventional ECG (orthogonal ± 12-lead) to obtain excellent diagnostic accuracies, albeit generally only for those conditions considered classically diagnosable by ECG, such as ventricular hypertrophy and previous infarction[38,39]. Our results therefore confirm Pipberger et al's suggestion that the diagnostic utility of resting ECG could be continuously improved through computer-automated multivariate analyses validated against ECG-independent diagnostic information. Our results also suggest that the use of 21^st^-century software technology can now extend the reach of resting ECG toward the detection of conditions previously thought not to be detectable by it, for example CAD without prior infarction. The basic premise of A-ECG is that a ~5-min resting 12-lead recording contains sufficient information, *if assiduously sought*, to allow gross detection of most cardiac pathology. Although the ECG equipment used in this study was "high fidelity," the best-performing A-ECG scores likely did not require such equipment and thus should also be derivable from many "standard-fidelity" ECG devices.

The present results suggest that with further validation, resting A-ECG might join other methods that are presently recommended [40,41] or suggested [42] as initial tests for individuals at intermediate pretest epidemiologic risk for CAD. The main advantages of A-ECG are that it can be performed rapidly and inexpensively, including in patients who are unable to exercise, and it does not expose patients to the potentially health-compromising effects of radiation. The convenience of A-ECG is also high in that the majority of individuals who are clinically discerned as being at intermediate pretest epidemiologic risk for CAD will likely have resting 12-lead ECGs anyway. Finally, A-ECG also has a multifunctional aspect in that it can potentially aid screening not only for CAD but also for LVH and LVSD.

Heart failure is an increasing and expensive problem worldwide. Because the adequate and timely treatment of LVSD can reduce mortality and frequency of hospitalizations,[43,44] it would be beneficial if a simple resting ECG could serve as a reasonably accurate initial screening test. Although our results corroborate the findings of others that a normal resting conventional 12-lead ECG has a very high NPV for LVSD (typically >95% in individuals with suspected heart failure in the general population),[4,5] conventional ECG's predictive value for LVSD is nonetheless limited by its simultaneously poor PPV (typically ≤35% in the same studies)[4,5]. Notably, in our study, the best secondary A-ECG scores nearly doubled the PPV of resting ECG for LVSD without compromising NPV. Given that the use of A-ECG therefore mitigates resting ECG's principal weakness in LVSD screening (poor PPV) and the fact that even conventional ECG alone sometimes outperforms other proposed modalities for LVSD screening such as B-type natriuretic peptide,[5] A-ECG might serve as a useful adjunct to conventional ECG and natriuretic peptides in heart failure screening, particularly for better guiding referrals to more definitive but costly echocardiography tests.

### Limitations

We did not nominally allow age, a continuous parameter that correlates with many ECG changes,[45] to be incorporated into A-ECG scores. We took this approach not only because the incorporation of age might ultimately compromise the ability of A-ECG to detect disease in younger individuals, but also because our principal aim was to compare the performance of A-ECG to that of optimized, strictly conventional ECG criteria that likewise do not incorporate age. While we are able to construct primary A-ECG scores that incorporate age and that, compared to the primary A-ECG scores described herein, have non-significantly increased accuracy in the full training set (where age differences between Healthy and Disease groups are greatest), these same age-incorporating scores also have non-significantly decreased accuracies in the arguably more important test set. Similarly, while we're also able to construct A-ECG scores on a gender-specific basis, doing so does not statistically significantly improve performance for either gender, neither in the training set nor in the test set. This finding may relate to the fact that the best performing non-gender specific scores all contained at least one parameter known to have higher values in men, for example spatial QRS-T angle,[46] balanced by at least one parameter known to have higher values in women, for example a measure of T-wave complexity[47]. Clearly, however, the use of gender-specific A-ECG scores validated in larger data sets might further optimize performance in the future.

Because our hypothesis involved assessing the *relative *performance of A-ECG versus conventional ECG and we were not able to assess coronary microvascular function, [48,49] we excluded from our "Healthy" groups asymptomatic diabetics, hypertensives and smokers as well as all individuals with angina or subclinical CAD (luminal stenoses <50% by catheterization). From the perspective of assessing *absolute *performance this might of course be construed as a limitation. To therefore further address this issue, we have proceeded to analyze the ECG data from these excluded higher risk individuals (N = 136; 55 ± 11 years, 51% females), the results revealing that just over one half (69/136) would have had positive optimized conventional ECG criteria for "Disease" and just over one-third (49/136; 51/136) positive full-disclosure and snapshot primary A-ECG scores, respectively. Thus, had we simply ignored any possible effect of subclinical CAD on the ECG (in spite of evidence to the contrary [50]) and just assigned these higher risk individuals to our "Healthy" group, not only would the specificity of all ECG testing been decreased, but the specificity of the primary A-ECG scores would also have been *further increased *relative to that of the optimized, strictly conventional ECG criteria. Additional studies, ideally using direct physiological assessment of coronary arteries [49] as the gold standard, are therefore required to determine whether any clinical importance should be attached to the modestly lower prevalence of A-ECG compared to conventional ECG abnormalities in these higher risk individuals.

Nearly all our patients with LVSD had experienced symptoms and had begun medical therapy prior to their ~5-min ECG tests. Therefore, although we demonstrated that A-ECG scores have better predictive value for medically-managed LVSD than do pooled criteria from the strictly conventional ECG, the ability of A-ECG to better predict pre-symptomatic LVSD was not directly tested and requires further study. Additional study limitations include the grouping together of CAD and LVH (keeping in mind that these conditions are commonly co-morbid plus the more important fact that a high suspicion of either during initial screening would prompt further characterization through imaging); the relatively small number of patients with isolated LVH in the test set; the absence of a larger prospectively studied test group without prior known Disease; and the use of multiple different imaging modalities. Finally, we have not studied the prognostic utility of A-ECG scores. Additional studies are therefore required to determine whether A-ECG scores can further augment the known prognostic utility of certain of their key constituent parameters [6,8,9,11,12,15].

## Conclusions

Resting 12-lead ECG tests that combine 7 or fewer advanced and conventional ECG parameters within computerized A-ECG scores are more accurate than optimized pooled criteria from the strictly conventional ECG in detecting obstructive CAD and concentric LVH and in screening for LVSD in individuals with known cardiac disease.

## Competing interests

All authors declare that they have no competing financial interests. TTS and JLD are inventors on NASA-owned patents involving high frequency QRS, an ECG technology that plays no role in A-ECG scoring. VS serves as an unpaid consultant to CardioSoft, a company that is pursuing a patent for real-time application of QT variability.

## Authors' contributions

TTS conceived the study and TTS, BV, MAR, MWB, TB, RD, SGW, TNM, RM, DJ, HA and OP acquired the data. TTS, WBK, AHF, ECG, JLD and VS conceived and/or implemented the various software-based advanced ECG techniques. AHF, TTS and MJH conceived and/or implemented the statistical methods. TTS drafted the manuscript with some assistance from AHF for the statistical section. All authors were involved in revising the manuscript critically and read and approved the final version.

## Pre-publication history

The pre-publication history for this paper can be accessed here:

http://www.biomedcentral.com/1471-2261/10/28/prepub

## Supplementary Material

Additional file 1 **(Supplemental Table 1): Performance of Selected Individual Conventional and Advanced ECG Parameters in the Training Set**. Click here for file

Additional file 2 **(Supplemental Table 2): Components and Coefficients of those Primary and Secondary A-ECG Logistic Scores generated from the Training Set that Performed Best in the Test Set**. Click here for file

## References

[B1] Sox HC Jr Garber AM Littenberg B The resting electrocardiogram as a screening test. A clinical analysis Ann Intern Med 1989 111 6 489 502 2528311 10.7326/0003-4819-111-6-489

[B2] Ashley EA Raxwal V Froelicher V An evidence-based review of the resting electrocardiogram as a screening technique for heart disease Prog Cardiovasc Dis 2001 44 1 55 67 10.1053/pcad.2001.24683 11533927

[B3] Levy D Labib SB Anderson KM Christiansen JC Kannel WB Castelli WP Determinants of sensitivity and specificity of electrocardiographic criteria for left ventricular hypertrophy Circulation 1990 81 3 815 820 2137733 10.1161/01.cir.81.3.815

[B4] Davie AP Francis CM Love MP Caruana L Starkey IR Shaw TR Sutherland GR McMurray JJ Value of the electrocardiogram in identifying heart failure due to left ventricular systolic dysfunction Br Med J 1996 312 7025 222 10.1136/bmj.312.7025.222 PMC2350031 8563589

[B5] Hedberg P Lonnberg I Jonason T Nilsson G Pehrsson K Ringqvist I Electrocardiogram and B-type natriuretic peptide as screening tools for left ventricular systolic dysfunction in a population-based sample of 75-year-old men and women Am Heart J 2004 148 3 524 529 10.1016/j.ahj.2004.03.034 15389243

[B6] Kardys I Kors JA van der Meer IM Hofman A van der Kuip DA Witteman JC Spatial QRS-T angle predicts cardiac death in a general population Eur Heart J 2003 24 14 1357 1364 10.1016/S0195-668X(03)00203-3 12871693

[B7] Fayn J Rubel P Pahlm O Wagner GS Improvement of the detection of myocardial ischemia thanks to information technologies Int J Cardiol 2007 120 2 172 180 10.1016/j.ijcard.2006.09.025 17184859

[B8] Rautaharju PM Kooperberg C Larson JC LaCroix A Electrocardiographic predictors of incident congestive heart failure and all-cause mortality in postmenopausal women: the Women's Health Initiative Circulation 2006 113 4 481 489 10.1161/CIRCULATIONAHA.105.537415 16449727

[B9] Yamazaki T Froelicher VF Myers J Chun S Wang P Spatial QRS-T angle predicts cardiac death in a clinical population Heart Rhythm 2005 2 1 73 78 10.1016/j.hrthm.2004.10.040 15851268

[B10] Schlegel TT Kulecz WB DePalma JL Feiveson AH Wilson JS Rahman MA Bungo MW Real-time 12-lead high frequency QRS electrocardiography for enhanced detection of myocardial ischemia and coronary artery disease Mayo Clin Proc 2004 79 339 350 10.4065/79.3.339 15008608

[B11] Okin PM Malik M Hnatkova K Lee ET Galloway JM Best LG Howard BV Devereux RB Repolarization abnormality for prediction of all-cause and cardiovascular mortality in American Indians: the Strong Heart Study J Cardiovasc Electrophysiol 2005 16 9 945 951 10.1111/j.1540-8167.2005.40808.x 16174013

[B12] Zabel M Malik M Hnatkova K Papademetriou V Pittaras A Fletcher RD Franz MR Analysis of T-wave morphology from the 12-lead electrocardiogram for prediction of long-term prognosis in male US veterans Circulation 2002 105 9 1066 1070 10.1161/hc0902.104598 11877356

[B13] Batdorf BH Feiveson AH Schlegel TT The effect of signal averaging on the reproducibility and reliability of measures of T-wave morphology J Electrocardiol 2006 39 3 266 270 10.1016/j.jelectrocard.2005.11.004 16529767

[B14] Berger RD Kasper EK Baughman KL Marban E Calkins H Tomaselli GF Beat-to-beat QT interval variability: novel evidence for repolarization lability in ischemic and nonischemic dilated cardiomyopathy Circulation 1997 96 5 1557 1565 9315547 10.1161/01.cir.96.5.1557

[B15] Piccirillo G Magri D Matera S Magnanti M Torrini A Pasquazzi E Schifano E Velitti S Marigliano V Quaglione R Barilla F QT variability strongly predicts sudden cardiac death in asymptomatic subjects with mild or moderate left ventricular systolic dysfunction: a prospective study Eur Heart J 2007 28 11 1344 1350 10.1093/eurheartj/ehl367 17101636

[B16] Vrtovec B Sinkovec M Starc V Radovancevic B Schlegel TT Coronary artery disease alters ventricular repolarization dynamics in type 2 diabetes Pacing Clin Electrophysiol 2005 28 Suppl 1 S178 181 10.1111/j.1540-8159.2005.00076.x 15683491

[B17] Starc V Schlegel TT Real-time multichannel system for beat-to-beat QT interval variability J Electrocardiol 2006 39 4 358 367 10.1016/j.jelectrocard.2006.03.004 16919668

[B18] Camm AJ Malik M Bigger JT Jr Breithardt G Cerutti S Cohen RJ Coumel P Fallen EL Kennedy HL Kleiger RE Lombardi F Malliani A Moss AJ Rottman JN Schmidt G Schwartz PJ Singer DH Heart rate variability. Standards of measurement, physiological interpretation, and clinical use. Task Force of the European Society of Cardiology and the North American Society of Pacing and Electrophysiology Eur Heart J 1996 17 3 354 381 8737210

[B19] Goldberger AL Amaral LA Hausdorff JM Ivanov P Peng CK Stanley HE Fractal dynamics in physiology: alterations with disease and aging Proc Natl Acad Sci USA 2002 99 Suppl 1 2466 2472 10.1073/pnas.012579499 11875196 PMC128562

[B20] Jain AK Duin RPW Mao J Statistical pattern recognition: a review IEEE transactions on pattern analysis and machine intelligence 2000 22 1 4 37 10.1109/34.824819

[B21] Persson E Carlsson M Palmer J Pahlm O Arheden H Evaluation of left ventricular volumes and ejection fraction by automated gated myocardial SPECT versus cardiovascular magnetic resonance Clin Physiol Funct Imaging 2005 25 3 135 141 10.1111/j.1475-097X.2005.00599.x 15888092

[B22] Cain PA Ahl R Hedstrom E Ugander M Allansdotter-Johnsson A Friberg P Arheden H Age and gender specific normal values of left ventricular mass, volume and function for gradient echo magnetic resonance imaging: a cross sectional study BMC Med Imaging 2009 9 2 10.1186/1471-2342-9-2 19159437 PMC2657902

[B23] Tragardh E Schlegel TT Carlsson M Pettersson J Nilsson K Pahlm O High-frequency electrocardiogram analysis in the ability to predict reversible perfusion defects during adenosine myocardial perfusion imaging J Electrocardiol 2007 40 6 510 514 10.1016/j.jelectrocard.2007.03.242 17531255

[B24] Lang RM Bierig M Devereux RB Flachskampf FA Foster E Pellikka PA Picard MH Roman MJ Seward J Shanewise JS Solomon SD Spencer KT Sutton MS Stewart WJ Recommendations for chamber quantification: a report from the American Society of Echocardiography's Guidelines and Standards Committee and the Chamber Quantification Writing Group, developed in conjunction with the European Association of Echocardiography, a branch of the European Society of Cardiology J Am Soc Echocardiogr 2005 18 12 1440 1463 10.1016/j.echo.2005.10.005 16376782

[B25] Okin PM Devereux RB Jern S Kjeldsen SE Julius S Dahlof B Baseline characteristics in relation to electrocardiographic left ventricular hypertrophy in hypertensive patients: the Losartan intervention for endpoint reduction (LIFE) in hypertension study. The Life Study Investigators Hypertension 2000 36 5 766 773 11082141 10.1161/01.hyp.36.5.766

[B26] Anderson WD Wagner NB Lee KL White RD Yuschak J Behar VS Selvester RH Ideker RE Wagner GS Evaluation of a QRS scoring system for estimating myocardial infarct size. VI: Identification of screening criteria for non-acute myocardial infarcts Am J Cardiol 1988 61 10 729 733 10.1016/0002-9149(88)91056-9 3354433

[B27] Kors JA van Herpen G Sittig AC van Bemmel JH Reconstruction of the Frank vectorcardiogram from standard electrocardiographic leads: diagnostic comparison of different methods Eur Heart J 1990 11 12 1083 1092 2292255 10.1093/oxfordjournals.eurheartj.a059647

[B28] Draper HW Peffer CJ Stallmann FW Littmann D Pipberger HV The Corrected Orthogonal Electrocardiogram and Vectorcardiogram in 510 Normal Men (Frank Lead System) Circulation 1964 30 853 864 14246330 10.1161/01.cir.30.6.853

[B29] Horinaka S Yamamoto H Tabuchi T Takada M Akabane T Onoda M Yagi S Ventricular gradient variability. New ECG method for detection of ischemic heart disease J Electrocardiol 1995 28 3 177 183 10.1016/S0022-0736(05)80255-9 7595119

[B30] Priori SG Mortara DW Napolitano C Diehl L Paganini V Cantu F Cantu G Schwartz PJ Evaluation of the spatial aspects of T-wave complexity in the long-QT syndrome Circulation 1997 96 9 3006 3012 9386169 10.1161/01.cir.96.9.3006

[B31] Solaimanzadeh I Schlegel TT Feiveson AH Greco EC DePalma JL Starc V Marthol H Tutaj M Buechner S Axelrod FB Hilz MJ Advanced electrocardiographic predictors of mortality in familial dysautonomia Auton Neurosci 2008 144 1-2 76 82 10.1016/j.autneu.2008.08.016 18851930

[B32] Ishizawa K Motomura M Konishi T Wakabayashi A High reliability rates of spatial pattern analysis by vectorcardiogram in assessing the severity of eccentric left ventricular hypertrophy Am Heart J 1976 91 1 50 57 10.1016/S0002-8703(76)80434-6 128284

[B33] Vacek JL Wilson DB Botteron GW Dobbins J Techniques for the determination of left ventricular mass by signal-averaged electrocardiography Am Heart J 1990 120 4 958 963 10.1016/0002-8703(90)90215-J 2145736

[B34] Starc V Schlegel TT The effect of aging and cardiac disease on that portion of QT interval variability that is independent of heart rate variability Computers in Cardiology 2008 35 315 317

[B35] Efron B The Jackknife, the Bootstrap and Other Resampling Models 1985 2 Bristol: Arrowsmith Ltd. (for Society for Industrial and Applied Mathematics (SIAM))

[B36] Fleiss JL Statistical Methods for Rates and Proportions 1981 2 New York: John Wiley & Sons

[B37] Wang W Davis CS Soong SJ Comparison of predictive values of two diagnostic tests from the same sample of subjects using weighted least squares Statistics in medicine 2006 25 13 2215 2229 10.1002/sim.2332 16220470

[B38] Kyle MC Klingeman JD Conrad JD Freis ED Pipberger HV A new microcomputer-based ECG analysis system Clin Cardiol 1983 6 9 447 455 10.1002/clc.4960060906 6688771

[B39] Pipberger HV McCaughan D Littmann D Pipberger HA Cornfield J Dunn RA Batchlor CD Berson AS Clinical application of a second generation electrocardiographic computer program Am J Cardiol 1975 35 5 597 608 10.1016/0002-9149(75)90044-2 1092149

[B40] Gibbons RJ Balady GJ Bricker JT Chaitman BR Fletcher GF Froelicher VF Mark DB McCallister BD Mooss AN O'Reilly MG Winters WL Gibbons RJ Antman EM Alpert JS Faxon DP Fuster V Gregoratos G Hiratzka LF Jacobs AK Russell RO Smith SC ACC/AHA 2002 guideline update for exercise testing: summary article. A report of the American College of Cardiology/American Heart Association Task Force on Practice Guidelines (Committee to Update the 1997 Exercise Testing Guidelines) J Am Coll Cardiol 2002 40 8 1531 1540 10.1016/S0735-1097(02)02164-2 12392846

[B41] Greenland P Bonow RO Brundage BH Budoff MJ Eisenberg MJ Grundy SM Lauer MS Post WS Raggi P Redberg RF Rodgers GP Shaw LJ Taylor AJ Weintraub WS Harrington RA Abrams J Anderson JL Bates ER Grines CL Hlatky MA Lichtenberg RC Lindner JR Pohost GM Schofield RS Shubrooks SJ Jr Stein JH Tracy CM Vogel RA Wesley DJ ACCF/AHA 2007 clinical expert consensus document on coronary artery calcium scoring by computed tomography in global cardiovascular risk assessment and in evaluation of patients with chest pain Circulation 2007 115 3 402 426 10.1161/CIRCULATIONAHA..107.181425 17220398

[B42] Naghavi M Falk E Hecht HS Jamieson MJ Kaul S Berman D Fayad Z Budoff MJ Rumberger J Naqvi TZ Shaw LJ Faergeman O Cohn J Bahr R Koenig W Demirovic J Arking D Herrera VL Badimon J Goldstein JA Rudy Y Airaksinen J Schwartz RS Riley WA Mendes RA Douglas P Shah PK From vulnerable plaque to vulnerable patient--Part III: Executive summary of the Screening for Heart Attack Prevention and Education (SHAPE) Task Force report Am J Cardiol 2006 98 2A 2H 15H 10.1016/j.amjcard.2006.03.002 16843744

[B43] Peacock WF Acute Emergency Department management of heart failure Heart Fail Rev 2003 8 4 335 338 10.1023/A:1026187013370 14574053

[B44] Pfeffer MA Braunwald E Moye LA Basta L Brown EJ Jr Cuddy TE Davis BR Geltman EM Goldman S Flaker GC Klein M Lamas GA Packer M Rouleau J Rouleau JL Rutherford J Wertheimer JH Hawkins CM Effect of captopril on mortality and morbidity in patients with left ventricular dysfunction after myocardial infarction. Results of the survival and ventricular enlargement trial. The SAVE Investigators N Engl J Med 1992 327 10 669 677 1386652 10.1056/NEJM199209033271001

[B45] Pipberger HV Goldman MJ Littmann D Murphy GP Cosma J Snyder JR Correlations of the orthogonal electrocardiogram and vectorcardiogram with consitutional variables in 518 normal men Circulation 1967 35 3 536 551 6021335 10.1161/01.cir.35.3.536

[B46] Scherptong RW Henkens IR Man SC Le Cessie S Vliegen HW Draisma HH Maan AC Schalij MJ Swenne CA Normal limits of the spatial QRS-T angle and ventricular gradient in 12-lead electrocardiograms of young adults: dependence on sex and heart rate J Electrocardiol 2008 41 6 648 655 10.1016/j.jelectrocard.2008.07.006 18817923

[B47] Taneja T Larsen J Goldberger J Kadish A Age, gender, and autonomic tone effects on surface electrocardiographic indices of ventricular repolarization Ann Noninvasive Electrocardiol 2001 6 4 290 297 10.1111/j.1542-474X.2001.tb00121.x 11686909 PMC7027736

[B48] Reis SE Holubkov R Conrad Smith AJ Kelsey SF Sharaf BL Reichek N Rogers WJ Merz CN Sopko G Pepine CJ Coronary microvascular dysfunction is highly prevalent in women with chest pain in the absence of coronary artery disease: results from the NHLBI WISE study Am Heart J 2001 141 5 735 741 10.1067/mhj.2001.114198 11320360

[B49] Kern MJ Lerman A Bech JW De Bruyne B Eeckhout E Fearon WF Higano ST Lim MJ Meuwissen M Piek JJ Pijls NHJ Siebes M Spaan JAE Physiological assessment of coronary artery disease in the cardiac catheterization laboratory: a scientific statement from the American Heart Association Committee on Diagnostic and Interventional Cardiac Catheterization, Council on Clinical Cardiology Circulation 2006 114 12 1321 1341 10.1161/CIRCULATIONAHA.106.177276 16940193

[B50] Mohlenkamp S Schmermund A Lehmann N Roggenbuck U Dragano N Stang A Moebus S Beck EM Schluter C Sack S Meinertz T Taylor A Jockel KH Erbel R Subclinical coronary atherosclerosis and resting ECG abnormalities in an unselected general population Atherosclerosis 2008 196 2 786 794 10.1016/j.atherosclerosis.2007.01.012 17350632

