# Supplementary material for: Accuracy of advanced versus strictly conventional 12-lead ECG for detection and screening of coronary artery disease, left ventricular hypertrophy and left ventricular systolic dysfunction
Source: BMC Cardiovasc Disord. 2010 Jun 16;10:28. doi: 10.1186/1471-2261-10-28 (PMC2894002; doi:10.1186/1471-2261-10-28)
Supplement: Additional file 1 — (Supplemental Table 1): Performance of Selected Individual Conventional and Advanced ECG Parameters in the Training Set. [file 1471-2261-10-28-S1.PDF]

Supplemental Table 1. Performance of Selected Individual Conventional and Advanced ECG Parameters in the Training Set

ECG Parameters	Disease Group (N=290)	Disease Subgroup A (LVEF<50 %, N=102)	Disease Subgroup B (LVEF≥50 %, N=188)	Healthy Group (N=418)	*Healthy vs. Disease*		*Disease A vs. Disease B*	
					P	****AU-ROC***	***P***	****AU-ROC***
CONVENTIONAL								
QRS axis (°)	25 ± 46	17 ± 54	30 ± 41	63 ± 26	<0.0001	0.78	0.033	0.58
P duration (ms)	114 ± 16	113 ± 17	114 ± 16	100 ± 14	<0.0001	0.78	NS	0.53
QTc interval (ms)	435 ± 37	449 ± 41	427 ± 32	403 ± 25	<0.0001	0.76	<0.0001	0.67
JTc interval (ms)	338 ± 36	349 ± 39	331 ± 33	311 ± 26	<0.0001	0.72	0.0001	0.64
Cornell prdt. (mV*ms)	158 ± 81	187 ± 86	143 ± 71	103 ± 52	<0.0001	0.71	<0.0001	0.66
Cornell voltage (mV)	1.6 ± 0.8	1.9 ± 0.8	1.5 ± 0.7	1.1 ± 0.5	<0.0001	0.71	0.0001	0.64
PR interval (ms)	169 ± 32	171 ± 32	168 ± 31	150 ± 20	<0.0001	0.70	NS	0.54
12-lead voltage (mV)	13.8 ± 4.8	13.4 ± 5.0	14.0 ± 4.7	16.2 ± 3.7	<0.0001	0.70	NS	0.55
QRS interval (ms)	98 ± 11	100 ± 12	96 ± 10	92 ± 9	<0.0001	0.64	0.003	0.60
S-L voltage (mV)	2.1 ± 1.0	2.0 ± 1.2	2.1 ± 0.9	2.2 ± 0.6	0.0003	0.58	NS	0.55
HR (beats/min)	68 ± 13	72 ± 14	66 ± 11	64 ± 11	0.001	0.57	0.0002	0.63
T axis (°)	40 ± 67	56 ± 75	31 ± 61	50 ± 18	NS	0.53	<0.0001	0.65
HF QRS ECG								
Kurtosis sum (units)	38 ± 6	35 ± 6	39 ± 6	44 ± 7	<0.0001	0.73	<0.0001	0.68
RAZ score (points)	53 ± 24	64 ± 23	49 ± 23	36 ± 19	<0.0001	0.71	<0.0001	0.64
RAZs (leads, of 12)	8.2 ± 2.8	9.3 ± 2.2	7.7 ± 2.8	6.3 ± 2.7	<0.0001	0.69	<0.0001	0.67
RMSv sum (μV)	31 ± 12	30 ± 12	32 ± 11	38 ± 13	<0.0001	0.65	NS	0.53
3D ECG								
Spatial areas								
SVG (mV*ms)	45.3 ± 22.1	35.1 ± 20.0	50.9 ± 21.3	91.4 ± 28.6	<0.0001	0.90	<0.0001	0.72
SM T (mV*ms)	40.5 ± 21.8	40.0 ± 21.8	40.6 ± 22.2	65.2 ± 23.6	<0.0001	0.80	NS	0.51
Z integral (mV*ms)	10.4 ± 10.2	18.0 ± 10.1	6.2 ± 7.5	0.9 ± 6.3	<0.0001	0.80	<0.0001	0.85
SM QRS (mV*ms)	33.1 ± 18.0	38.5 ± 21.8	30.2 ± 14.9	32.8 ± 12.1	NS	0.54	0.0002	0.63
Spatial angles								
Mean QRS-T (°)	99 ± 42	132 ± 29	84 ± 39	43 ± 21	<0.0001	0.87	<0.0001	0.80
Peaks QRS-T (°)	72 ± 46	106 ± 45	57 ± 39	25 ± 15	<0.0001	0.84	<0.0001	0.77
SVG elevation (°)	30 ± 25	35 ± 25	27 ± 24	39 ± 8	<0.0001	0.60	0.004	0.60
Spatiotemporal								
SVG CV (ms)	16.3 ± 14.1	21.4 ± 18.2	14.1 ± 10.9	5.7 ± 4.3	<0.0001	0.86	0.0008	0.62
SM T CV (ms)	15.4 ± 12.5	14.1 ± 9.9	16.3 ± 13.7	6.7 ± 5.4	<0.0001	0.79	NS	0.53
Spatial VAT (ms)	45 ± 9	52 ± 9	43 ± 7	43 ± 6	<0.0001	0.61	<0.0001	0.75
SM QRS CV(ms)	4.3 ± 2.4	3.4 ± 2.0	4.8 ± 2.4	3.8 ± 1.9	0.017	0.55	<0.0001	0.69
Late Potentials								
fQRSd (ms)	97 ± 12	102 ± 13	95 ± 11	94 ± 9	0.001	0.58	0.0001	0.64
RMS40 (μV)	42 ± 30	45 ± 33	41 ± 29	48 ± 32	0.026	0.55	NS	0.52
LAS40 (ms)	29 ± 11	29 ± 12	29 ± 10	30 ± 8	NS	0.51	NS	0.51
COMPLEXITY								
QRS complexity								
QRS DPV (Ln μV)	9.82 ± 0.34	9.89 ± 0.37	9.78 ± 0.33	9.91 ± 0.27	<0.0001	0.60	0.02	0.59
QRS NDPV (Ln μV)	6.89 ± 0.46	7.06 ± 0.53	6.80 ± 0.39	6.98 ± 0.42	0.0023	0.57	0.0001	0.64
QRS IDR (%)	4.67 ± 3.54	4.73 ± 4.24	4.61 ± 3.21	3.86 ± 2.37	0.04	0.55	NS	0.54
QRS rWR (%)	0.24 ± 0.21	0.31 ± 0.31	0.21 ± 0.15	0.22 ± 0.15	NS	0.51	0.001	0.63
QRS PCA ratio (%)	45.9 ± 20.6	39.9 ± 21.1	48.6 ± 19.7	46.1 ± 18.2	NS	0.50	0.002	0.63
T complexity								
T IDR (%)	2.27 ± 3.62	2.72 ± 3.71	2.01 ± 3.47	0.39 ± 0.80	<0.0001	0.84	0.002	0.61
T rWR (%)	0.09 ± 0.28	0.12 ± 0.30	0.07 ± 0.27	0.01 ± 0.02	<0.0001	0.81	0.005	0.60

T DPV (Ln µV)	8.88 ± 0.50	8.79 ± 0.54	8.93 ± 0.47	9.39 ± 0.41	<0.0001	0.79	0.003	0.60
T PCA ratio (%)	27.9 ± 18.3	32.7 ± 19.5	25.4 ± 16.9	14.4 ± 6.8	<0.0001	0.75	0.001	0.61
T NDPV (Ln µV)	5.11 ± 0.49	5.13 ± 0.58	5.10 ± 0.45	5.00 ± 0.37	0.007	0.56	NS	0.52
RRV								
LFP (Ln ms^2/Hz)	4.2 ± 1.8	3.2 ± 2.2	4.6 ± 1.4	6.2 ± 1.2	<0.0001	0.84	<0.0001	0.66
HFP (Ln ms^2/Hz)	4.0 ± 1.7	3.3 ± 1.8	4.3 ± 1.5	6.2 ± 1.5	<0.0001	0.84	0.0001	0.64
RMSSD (ms)	20 ± 14	15 ± 12	22 ± 15	50 ± 38	<0.0001	0.83	0.0003	0.63
SD1 (ms)	14 ± 10	11 ± 8	16 ± 10	36 ± 27	<0.0001	0.82	0.0004	0.62
SDNN (ms)	29 ± 18	24 ± 19	31 ± 17	59 ± 32	<0.0001	0.82	0.0009	0.62
TFP (Ln ms^2/Hz)	5.8 ± 1.5	5.2 ± 1.8	6.1 ± 1.2	7.5 ± 1.1	<0.0001	0.82	0.0001	0.64
SD2 (ms)	38 ± 24	32 ± 25	41 ± 22	75 ± 38	<0.0001	0.82	0.001	0.62
Alpha 2 (units)	1.05 ± 0.22	1.10 ± 0.26	1.03 ± 0.20	0.83 ± 0.22	<0.0001	0.76	0.02	0.58
Alpha 1 (units)	1.04 ± 0.35	0.96 ± 0.36	1.08 ± 0.35	1.02 ± 0.30	NS	0.53	0.05	0.58
LFP/HFP	2.6 ± 4.9	2.0 ± 2.9	2.8 ± 5.6	1.8 ± 2.0	NS	0.52	NS	0.57
QTV								
QTVI (II, units)	-1.00 ± 0.67	-0.66 ± 0.75	-1.14 ± 0.57	-2.00 ± 0.35	<0.0001	0.92	<0.0001	0.66
QTVI (V5, units)	-1.13 ± 0.69	-0.77 ± 0.72	-1.28 ± 0.61	-1.97 ± 0.32	<0.0001	0.89	<0.0001	0.67
RMSSD (II, ms)	5.9 ± 4.2	7.0 ± 5.1	5.4 ± 3.6	2.0 ± 1.3	<0.0001	0.89	0.02	0.58
IUQTV (V5, units)	1.01 ± 0.59	1.08 ± 0.61	0.97 ± 0.58	0.23 ± 0.43	<0.0001	0.87	0.05	0.58
QTVI (E1, units)	-1.38 ± 0.70	-1.13 ± 0.82	-1.49 ± 0.61	-2.06 ± 0.35	<0.0001	0.83	0.001	0.62
IUQTV (E1, units)	0.85 ± 0.52	0.91 ± 0.53	0.82 ± 0.52	0.25 ± 0.44	<0.0001	0.82	0.05	0.58
IUQTV (II, units)	1.16 ± 0.53	1.14 ± 0.5	1.17 ± 0.52	0.52 ± 0.53	<0.0001	0.81	NS	0.51
RMSSD (V5, ms)	4.8 ± 4.2	5.7 ± 4.5	4.3 ± 3.9	2.0 ± 1.0	<0.0001	0.79	0.001	0.62
SDNN (II, ms)	5.0 ± 3.2	5.9 ± 4.1	4.5 ± 2.6	2.6 ± 1.4	<0.0001	0.78	0.03	0.58
RMSSD (E1, ms)	3.5 ± 4.0	4.2 ± 5.5	3.2 ± 2.9	1.7 ± 1.6	<0.0001	0.71	NS	0.55
SDNN (V5, ms)	4.4 ± 3.3	5.1 ± 3.6	4.1 ± 3.1	2.7 ± 1.3	<0.0001	0.69	0.009	0.59
SDNN (E1, ms)	3.4 ± 3.0	3.7 ± 3.9	3.2 ± 2.4	2.5 ± 1.5	0.0001	0.59	NS	0.53

All values are mean ± SD. See main text for relevant methodological citations. Individual P and AU-ROC (Area under the Receiver Operating Characteristic curve) values are strictly for descriptive purposes, with the former based on the nonparametric Wilcoxon sign ranked test. HR, Heart rate; S-L, Sokolow-Lyon; HF: high frequency (150-250 Hz); RAZ, Reduced Amplitude Zone; RMSv, root-mean-square voltage; SVG, spatial ventricular gradient; SM, spatial mean; Z integral, the total integral of the Z-lead QRS complex above 5 Hz; CV, coefficient of variation; VAT, ventricular activation time; fQRSd, filtered QRS duration (40-250 Hz); RMS40 and LAS40, the RMSv of the terminal 40 ms and the duration of low amplitude (<40 μV) signal in the terminal filtered (40-250 Hz) QRS complex, respectively; QRS IDR and T IDR: the "intradipolar ratio" of the QRS and T waves, respectively calculated as the product of the second and third singular values of the given waveform (QRS or T) divided by the square of the first singular value of the same waveform, multiplied by 100%; QRS rWR and T rWR: the "relative residua" of the QRS and T waveforms, respectively; QRS PCA and T PCA ratios: "principal component analysis" ratios of the QRS and T waveforms, respectively; QRS and T DPV and NDPV: the dipolar and nondipolar voltages, respectively, of the QRS and T waveforms, respectively, calculated as the sum of the first through third (DPV) and fourth through eighth (NDPV) singular values of the given waveform; respectively. RRV: R-wave to R-wave interval variability; LFP, HFP and TFP: low (0.04-0.15 Hz), high (0.15-0.4 Hz) and total (0.0-0.4 Hz) frequency powers, respectively; SDNN, standard deviation of normal-to-normal intervals; SD1, SD2: short- and long-term standard deviations, respectively, from the Poincarė RR-interval plot; RMSSD, the root-mean-square of differences between consecutive intervals; Alpha 1, Alpha 2: fractal parameters of RRV derived from detrended fluctuation analysis. QTV, QT interval variability, with results shown for standard leads II and V5 and for the first eigenvector (E1) derived from singular value decomposition; QTVI, QT variability index; IUQTV, index of unexplained QTV.

*For comparison with the individual parameters shown, the best performing primary and secondary A-ECG scores had AU-ROCs exceeding 0.98 and 0.91, respectively, in the same training set.
