# Supplementary material for: Accuracy of advanced versus strictly conventional 12-lead ECG for detection and screening of coronary artery disease, left ventricular hypertrophy and left ventricular systolic dysfunction
Source: BMC Cardiovasc Disord. 2010 Jun 16;10:28. doi: 10.1186/1471-2261-10-28 (PMC2894002; doi:10.1186/1471-2261-10-28)
Supplement: Additional file 2 — (Supplemental Table 2): Components and Coefficients of those Primary and Secondary A-ECG Logistic Scores generated from the Training Set that Performed Best in the Test Set. [file 1471-2261-10-28-S2.PDF]

Supplemental Table 2. Components and Coefficients of those Primary and Secondary A-ECG Logistic Scores generated from the Training Set that Performed Best in the Test Set

A-ECG score component	Coefficients of the 7-Parameter Primary ("Healthy vs. Disease") A-ECG score requiring ~5-min "full disclosure" ECGs	Coefficients of the 7-Parameter Primary ("Healthy vs. Disease") A-ECG Score obtainable from 10-sec "snapshot" ECGs	Coefficients of the 5-Parameter Secondary ("Disease with vs. without LVSD") A-ECG Score obtainable from 10-sec "snapshot" ECGs
CONVENTIONAL			
P duration (ms)	+0.0880089	+0.0947843	
QRS axis (°)	-0.0225851	-0.0300893	
12-lead voltage (Ln mV)			–3.985739
3D ECG			
Spatial areas			
*SVG-SM QRS (mV*ms)†		-0.7675504	
Z integral (mV*ms)			+0.1490614
Spatial angles			
Mean QRS-T (°)	+0.0333293		+0.0172501
Peaks QRS-T (°)		+0.0352906	
SVG elevation (°)	-0.0555467	-0.0628153	
COMPLEXITY			
QRS complexity			
†QRS NDPV (Ln µV)			+2.249326
T complexity			
T IDR (Ln %)	+1.301927		
T PCA (Ln %)		+1.665364	
T DPV (Ln µV)			-1.26528
QTV			
QTVI (II, units)	+2.685218		
IUQTV (V5, units)	+1.276661		
Constant	-4.505596	-10.094	+1.672794

A-ECG, advanced electrocardiography; LVSD, left ventricular systolic dysfunction; SVG, spatial ventricular gradient; SM, spatial mean; Z integral, the total integral of the Z-lead QRS complex filtered above 5 Hz; QRS NDPV, nondipolar voltage of the QRS wave; T IDR, intradipolar ratio of the T wave, calculated as the product of the second and third singular values divided by the square of the first singular value of the signal-averaged T wave, multiplied by 100%; T DPV, dipolar voltage of the T wave; QTV, QT interval variability; QTVI, QT variability index (in lead II); IUQTV, index of unexplained QTV (in lead V5).

*This composite parameter, which represents, in units of mV*ms, the arithmetic subtraction of the SM QRS area from the SVG area, is akin but not equivalent to the SM T area.

†Adequate reproducibility and reliability of results from this parameter in 10-sec snapshot ECG recordings remains to be proven but is assumed based on its use with snapshot recordings in the recent publications of Rautaharju et al. See for example reference [8] in the main text.
